# Preparation and characterization of homogeneous and enhanced casein protein-based composite films via incorporating cellulose microgel

**DOI:** 10.1038/s41598-018-37848-1

**Published:** 2019-02-04

**Authors:** Yijun Yao, Hongru Wang, Ruirui Wang, Yong Chai

**Affiliations:** College of Bioresources Chemical and Materials Engineering, Shaanxi University of Science and Technology, Xi’an, 710021 China

## Abstract

Gelatin-coupled cellulose (GCC) microgel with whisker-like structure is prepared and used to incorporate into casein (CA) matrix to construct reinforced CA-based composite films by solution casting. The GCC microgel has excellent dispersibility and stability in water, which contributes to the hydrophobicity and significantly reduces the moisture absorption of the composite films, as well as a decrease in the water vapor permeability with an increase of GCC content at different relative humidity is also observed. Compared with pure casein material, the resultant CA-based composite films show more homogeneous and dense cross-sectional structure, and the cleavage temperature of the hydrogen bonds increases by 16 °C. In particular, their tensile strength and Young’s modulus increase by 6 and 3.5 times, respectively. These indicators are superior to that of the nanoparticle enhanced CA-based composite film. Moreover, the light transmittance of the CA-based films at 550 nm is about 88% when GCC content is higher than 9%. The above results could be attributed to the strong hydrogen bonds formed between GCC components and CA matrix, as further confirmed by fourier transform infrared spectra and X-ray diffraction analysis.

## Introduction

In order to alleviate the increasingly severe energy depletion and environmental pollution caused by petroleum based polymers, natural materials have become the hot topic for research due to their renewability, biocompatibility and biodegradability^1–3^. At the same time, special attention is paid to the demand for natural polymer coatings with strong moisture resistance and mechanical properties^4^. As a leather finishing material, casein has the advantages of high temperature resistance, strong adhesion, and excellent hygienic property^5^. It is extracted from milk and compose of four different proteins, *α*-(s1)- and *α*-(s2)-caseins, *β*-casein, and *κ*-casein^6^. However, casein films are always inflexible because of the presence of many secondary bonds in casein chains, resulting in poor activity and extensibility of the peptide chains. In addition, due to a variety of hydrophilic groups including carboxyl group (-COOH), amino group (-NH_2_), and imino group (-NH-) in casein structure, casein films are sensitive to humidity and easily destroyed under wet conditions which limit its further application.

Chemical modification of casein has been performed for satisfying the important properties of casein for specific application. Although vinyl monomers (e.g. methyl methacrylate, butyl acrylate, and caprolactam)^7–9^, crosslinking agent^10–13^ or polyurethane^14,15^ have been grafted to the α-C or N of casein structure, which can introduce new components and block some hydrophilic groups of casein structure, resulting in high hydrophobicity and mechanical tensile strength and elasticity of the casein films. However, it involves harsh reaction conditions and environmental concerns. Transglutaminase (TG) from microorganisms is non-toxic and feasible, and also produces cross-linking of protein to improve the thermo-stability of casein; this is done by catalyzing acyl transfer reactions^16–18^. However, enzyme has uncontrollable features inherently resulting in difficulty of controlling the reaction.

In recent years, casein-based composite films with higher performances have been prepared through physical incorporation of nanoparticles into casein system without extra chemicals^19–25^. Nanotechnology is a promising strategy to improve water resistance, mechanical, and hygienic properties of the obtained casein-based composite films, unfortunately, most of the nanoparticles are easily aggregated in the matrix, thus decreasing the reinforcement effect and affecting their special properties. Therefore, further modification of nanoparticles to ensure the uniform dispersion in polymer matrix is always needed^26^, which is obviously a complex and time-consuming process.

Microgels, colloids of physically/chemically cross-linked polymer network structure internally, have attracted wide interest for application in enhancing the mechanical property and durability of polymer films, because their high dispersibility and stability, micro/nano structures and high specific surface area^27^. In our previous study, we have synthesized a self-dispersed gelatin-coupled cellulose (GCC) microgel system in NaOH/urea aqueous media, and we found that the particle size of the microgel was nanoscale with whisker-like structure, and the film obtained from the microgel system exhibited relatively strong water resistance, thermal stability and mechanical property^28^. Moreover, in addition to the characteristics of intrinsic high elastic modulus and web-like structure of cellulose itself existed in GCC backbone, a large number of similar groups in gelatin and casein proteins provide an opportunity to enhance the phase of nano-gel compatibility. Inspired by these interesting phenomena, in this work, the GCC microgel is used as a reinforced material to incorporate into casein matrix to fabricate the homogeneous and enhanced casein-based composite films through the solution casting, thereby evaluating the effect of GCC microgel on the structure and properties of the resultant casein films including morphologies, structural changes, transparency, thermal stability, moisture absorption, water vapor permeability, and mechanical properties.

## Experimental

### Materials

Casein (protein content ≥87.5%) was obtained from Beijing Aobox biotechnology Co., Ltd. (Beijing, China), its weight molecular(Mw) was 768~65680 Da which was determined by GPC (see Fig. S1 and Table S1). Gelatin was supplied by YI Weilong Biotechnology Co., Ltd. (Xiamen, China), its weight molecular was 882~63188 Da. Cellulose (cotton linter pulps, α-cellulose ≥95%) was provided by Hubei Chemical Fiber Co. Ltd. (Xiangfan, China). Epichlorohydrin (ECH) (analytical grade, liquid, 1.18 g/mL), NaOH and urea were purchased from Tianli Chemical Reagents Ltd. (Tianjin, China). All the chemicals were analytical grade and used without further purification.

### Fabrication of self-dispersed gelatin-coupled cellulose (GCC) microgel

GCC microgel was obtained according to a previously described method^28^. Briefly, 3 wt% cellulose-NaOH/urea/H_2_O (7/12/81, by weight) solution was prepared, and then the cellulose solution was blend with a 10 wt% gelatin aqueous solution and a coupling agent ECH to obtain a raw material GCC product. Any unreacted ECH, NaOH, urea and by-products formed during this process can be removed through dialysis against deionized water for a week. The molecular weight cut off threshold of the dialysis film used is 100 kDa. Finally, after dialysis, a certain amount of deionized water was added into the raw GCC product under vigorous stirring for 30 min, and then separated into layers after sediment for overnight. The bottom layer contained un-dissolved cellulose and its coupled product, and the upper layer was GCC microgel. Based on our previous studies, we controlled cellulose/gelatin with a ratio of 8/2 by weight and the molar ratio of ECH to anhydroglucose unit of cellulose was 2:1 throughout the system^28^.

### Preparation of casein-based composite films

10 grams of casein powders were dispersed into 85 grams of deionized water, then 5 grams of NaOH aqueous solution (5 wt%) were added to dissolve casein with constant stirring at 50 °C for 45 min to obtain 10 wt% casein aqueous solution. The above GCC microgel was mixed mechanically with casein solution at 50 °C, and a series of GCC/casein blend solutions with different of GCC contents of 0, 3, 6, 9, 12, and 15 wt% were prepared, respectively. The blended solution was poured into a PTFE plastic plate and dried at a constant temperature of 25 °C to obtain a corresponding film, coded as CA, GCC-CA-3%, GCC-CA-6%, GCC-CA-9%, GCC-CA-12%, and GCC-CA-15%, respectively. The pure casein film was used as a blank control. The thickness of the CA-based films was about 0.15 mm.

### Characterization of GCC microgel and casein-based composite films

The molecular weights of samples were measured on a water-based gel permeation chromatography (GPC) system (Waters 2695 GPC). Samples were diluted to 3 mg/mL with 0.1 M NaNO_3_, and obtained by filtering with 0.45 μm RC filter membrane. The average particle size and size distribution of GCC microgel was measured using a Malvern Zetasizer ZS Instrument (Dynamic light scattering, DLS) (NANO-ZS90, Britain).

To determine the gelatin content (*W*_*Pro*_) in the GCC film, the N content (*W*_*N*_) in the microgel film was measured by using element analysis instrument (Vario EL III, Germany). The *W*_*Pro*_ value of microgel film can be calculated by^29^:

$${W}_{Pro}={W}_{N}\times 6.25$$

Fourier transform infrared (FTIR) spectroscopy of the CA-based films was directly measured on FTIR spectrometer (Vertex 70, BRUKER, Germany) in the range of 4000–500 cm^−1^.

The morphologies of GCC microgel and CA-based films were examined with a scanning electron microscopy (SEM) (FEI Quanta 600 FEG, America). A drop of GCC microgel solution was dripped onto the copper sheet, and then the morphology was observed by spraying gold after drying at room temperature. The CA-based films were frozen and sliced in liquid nitrogen to obtain the cross sections, then the cross sections were coated with gold and observed their morphologies. Wide-angle X-ray diffraction (XRD) measurement was carried out through using an XRD diffractometer (D8 Advance, Bruker, Germany). The patters with Cu-Kα radiation (λ = 0.15406 nm) at 40 kV and 40 mA were recorded over the region of 2θ from 5° to 55°.

The light transmittance of the composite films was measured using an UV-visible spectroscopy (Cary 5000, Agilent, USA) over a wavelength range from 200 to 800 nm with air as the background. The thermal stability of CA-based films was characterized using DSC analyzer (STA449, Bruker, Germany). Approximately 3~10 mg of the powder samples was used for each test, the test was conducted in a nitrogen atmosphere, and the test temperature ranged from 30 to 700 °C at a gradual increase rate of 10 K⋅min^−1^.

### Moisture absorption and water vapor permeability (WVP) analysis

The moisture absorption of the films was measured according to the previously reported method^30^. A piece of dry film (30 mm × 30 mm) with the initial weight (*W*_0_) was stored in a desiccator with saturated NaCl solution (75% RH) for 72 h; the final wet film weight was recorded as *W*_1_. The equilibrium moisture absorption (*W*, %) of composite films can be calculated using the following equation.

$$W=\frac{{W}_{1}-{W}_{0}}{{W}_{0}}\times 100{\rm{ \% }}$$

WVP of CA-based composite film was measured by permeability cup method according to the ASTM E96-95 standard. First, put 3 g anhydrous CaCl_2_ into a permeability cup with area of 10 cm^2^. Then seal the composite film at the edge of the cup using molten paraffin. Keep the cup in a desiccator for 24 h. The temperature and humidity of the desiccator were modulated by two kinds of different saturated salt aqueous solutions (MgCl_2_, 31% of RH at 25 °C; NaCl, 75% of RH at 30 °C) respectively. The WVP values were calculated using the following equation.

$${\rm{W}}{\rm{V}}{\rm{P}}[{\rm{m}}{\rm{g}}/(10{{\rm{c}}{\rm{m}}}^{2}\cdot 24{\rm{h}})]={m}_{1}-{m}_{2}$$

where *m*_1_ and *m*_2_ are the total mass of permeability cup, CaCl_2_, and composite film before and after treatment for 24 h respectively.

### Surface energy analysis

The surface energy analysis of CA-based composite films was determined by the measurement of water contact angles. Five parallel measurements were carried out for each film by using a contact angle analyzer (KRUSS, Germany).

### Mechanical testing

The mechanical properties of the prepared CA-based films were measured on an electronic tensile tester (AI-7000S, China) at a speed of 5 mm⋅min^−1^ according to ASTM/D638-91. Each film of 50 mm × 5 mm (analyzed area = 20 mm × 5 mm) was stored for a week at RH of 50% and 25 °C prior to the measurement.

## Results and Discussion

### Preparation of self-dispersed GCC microgel

**Figure 1:**
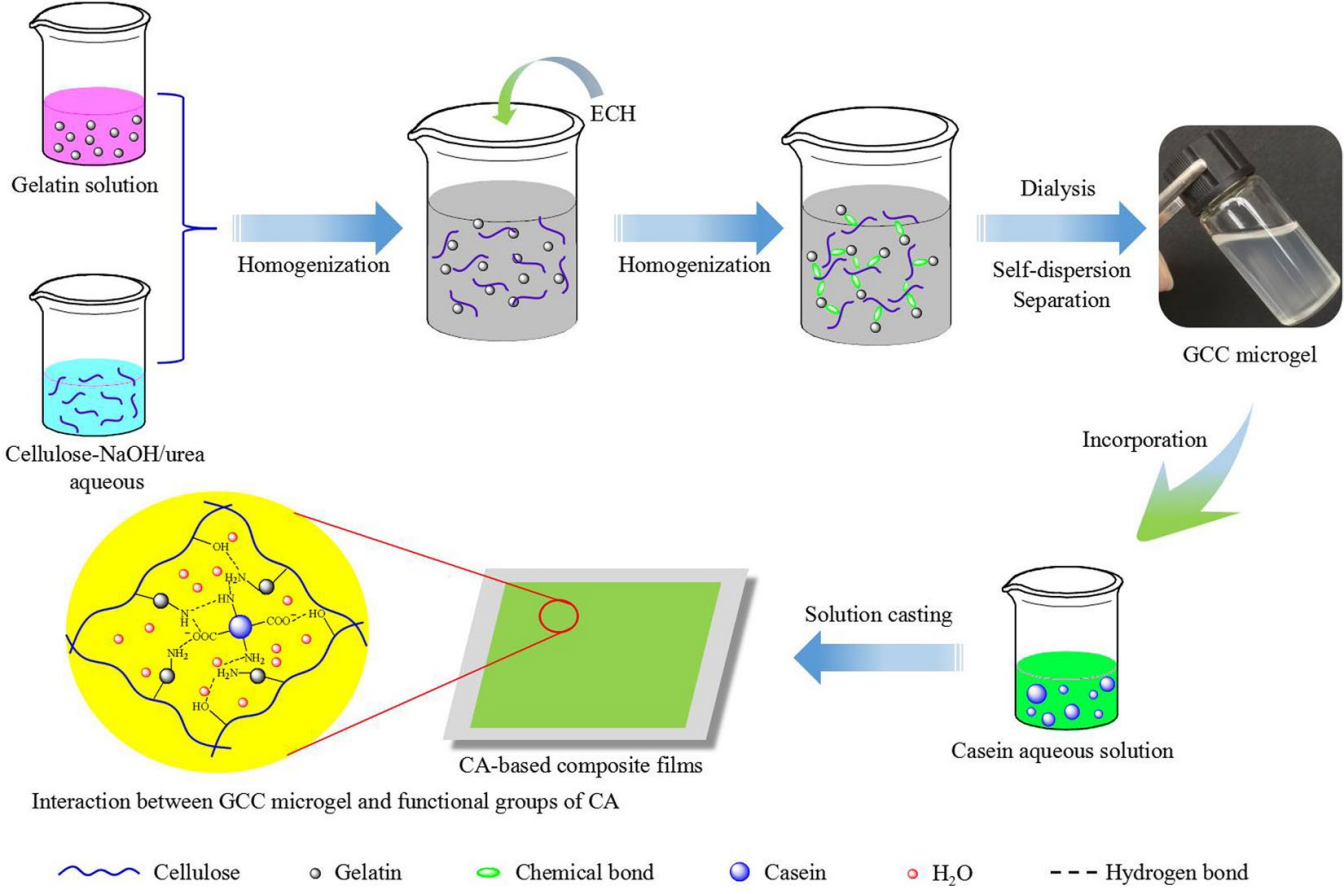
Process for the preparation of the CA-based composite films.

The GCC microgel is prepared by a one-step synthesis as illustrated in Fig. 1. Firstly, the homogeneous system of gelatin solution and cellulose-NaOH/urea aqueous solution are formed, then the coupling agent ECH is added dropwisely into the blend solutions with stirring for 2 h, finally, the GCC microgel are obtained by simple dialysis, self-dispersion and separation process. It is obvious that after adding gelatin and ECH, the cellulose-NaOH/urea system became very complex. Among them, gelatin and gelatin, cellulose and cellulose, and gelatin and cellulose could be coupled by ECH, respectively. To prove the reaction in this complex system was performed as expectation and the final product included only GCC microgel. The molecular weight of the possible reaction products was parallel measured firstly by GPC (see Table S2). As shown in Table S2, The average molecular weights (Mw) of gelatin, the gelatin treated in NaOH/urea aqueous media at the absence of cellulose and ECH (Gel-NaOH/urea), ECH-coupled gelatin in NaOH/urea aqueous media at the absence of cellulose (C-gel-NaOH/urea), and GCC are 0.88~63.19, 0.60~2.21, 0.78~10.62 and 636.60 kDa, respectively. Obviously, the molecular weight of gelatin significantly decreased after treated in NaOH/urea aqueous media due to hydrolysis, however, the molecular weight of hydrolyzed gelatin increased from 2.21 to 10.62 kDa via homocoupling reaction of ECH. Moreover, it was clear that GCC exhibited a higher molecular weight of 636.60 kDa than that of gelatin and C-gel-NaOH/urea, indicating new polymer is formed from coupling polymerization of cellulose and gelatin. The MWCO of the dialysis membrane used in this work is 100 kDa, therefore it is believed that the gelatin, gelatin hydrolysate and its coupling product in the resultant blend product can be removed during dialysis process, and the unreacted cellulose and its coupling products would form precipitation, to get the GCC microgel as desired by separation. As exhibited in Table S3, the protein content in GCC microgel and the corresponding yield is 18.69% and 21.3%, respectively. We successfully achieved self-dispersed cellulose microgel via coupling reaction, mainly due to the coupling agent ECH could react with -OH and -NH_2_ to form a new functional group under the alkaline conditions^31–33^, causing the strong H-bond within cellulose matrix can be restrained by the effect of steric hindrance and hydrophilic macromolecules, which effectively prevent the rapid reaggregation of cellulose in subsequent dialysis process.

The FTIR spectra of cellulose, gelatin, and GCC microgel are shown in Fig. S2. Compared to gelatin spectrum, the FTIR spectra of GCC apparently have two new peaks at 3343 and 1030 cm^−1^ attributed to O-H and C-O-C groups of cellulose^34,35^, respectively, which demonstrates the peptide chains of gelatin are successfully coupled with the glucose chain of cellulose. Because the undissolved cellulose and its coupled products could form precipitation and be removed after separation, the attached cellulose peaks on gelatin molecules could only be derived from the characteristic peaks of GCC. Moreover, in comparison to the cellulose spectrum, the intensities of the peaks for the C-O-C (1024–1104 cm^−1^) and C-H (2896 cm^−1^) increased obviously in GCC microgel, this increase was caused by the coupling agent ECH can provide abundant C-O-C and C-H groups^36^. FTIR confirmed successful coupling interactions occurred between cellulose and gelatin during the fabrication process.

**Figure 2:**
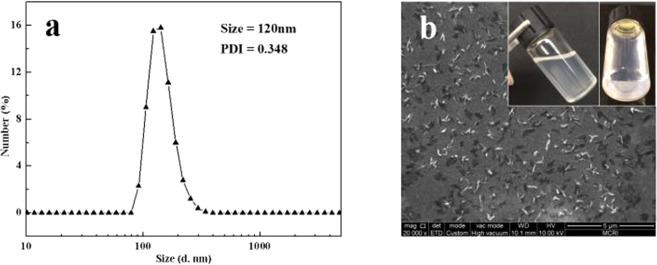
The particle size distribution (**a**), and SEM image (**b**) of GCC microgel. (In the illustration of (**b**), the left side image is GCC microgel solution, and the right side image is the bottom of the GCC microgel after standing for 1 month).

Figure 2a~b shows the particle size and the image of GCC microgel, respectively. As shown in Fig. 2a, the most of microgel size is concentrated around 120 nm, and the obtained GCC microgel presents “needle-like” shape with uniform distribution and its average length and diameter are 120 nm and 10 nm, respectively (Fig. 2b). From the illustrations, we can observe that the microgel is translucent and no sediment is found on the bottom of the small bottle after standing for 1 month. These results indicate that the GCC microgel has excellent dispersibility and stability in water. This could be attributed to the massive polar groups and covalently bonded web-like structure in GCC, which prevent the microgel from aggregation. Consequently, from the results of element analysis, GPC, FTIR, and DLS, the GCC microgel has been successfully prepared.

### Structure and compatibility of CA-based composite films and the interactions between GCC microgel and casein

**Figure 3:**
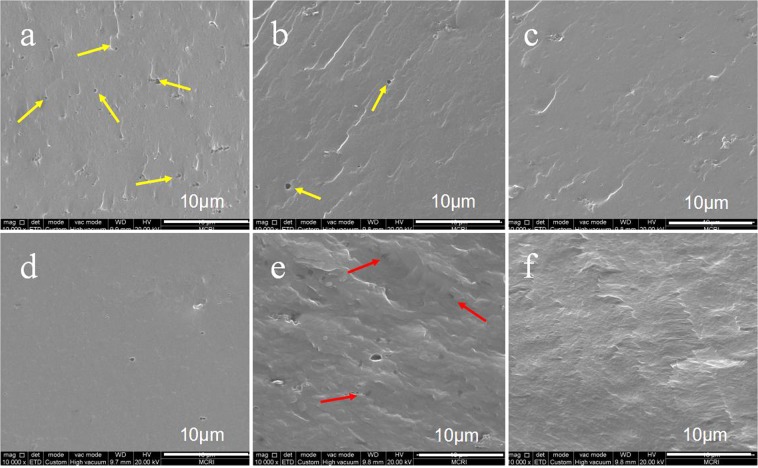
SEM images of the cross sections of CA (**a**), GCC-CA-6% (**b**), GCC-CA-9% (c), GCC-CA-12% (**d**), GCC-CA-15% (**e**), and GCC (**f**) films. *Note the yellow arrows designate the pores of casein film*, *and the red arrows point to GCC microgel*.

The process for the preparation of the CA-based composite films is shown in Fig. 1. In order to study the interaction between GCC microgel and casein matrix, the liquid nitrogen cross section of the CA-based composite film and GCC microgel film are observed using SEM, as shown in Fig. 3. The pure casein film has a rough cross section and presents a certain number of nano-scale pores as shown in Fig. 3a. The rough structure probably results from the massive polar, nonpolar, and the peptide chain self-aggregation during the drying process^37^, and the porous structure should be the pores produced from casein emulsion during the process of film formation, mainly ascribes to the discontinuous film-forming characteristic of casein itself^22^. In contrast, the cross section of GCC microgel film is compact and homogenous (Fig. 3f). With increasing the GCC microgel contents in CA-based composite films, the pores of casein are filled up gradually and the section morphologies become smooth (Fig. 3b–d), indicating the continuities and compactness of the composite films are enhanced. This is due to multi-hydroxyl, amino and imino groups on the GCC surface, enhancing the interfacial compatibility between CA matrix and GCC^38^. However, the section morphology becomes rough again, and several black spots but no obvious aggregation appear onside the CA-based composite film when the GCC microgel content is further increased to 15% (Fig. 3e), which could be attributed to the heterogeneous combination of GCC microgel with the CA matrix, and excessive GCC microgel addition in the CA matrix would lead to the phase separation^37^, thus affect the homogeneity of CA-based film. The cross-sectional morphologies of the composite films determine their hydrophobic, mechanical, light transmittance, and water vapor permeability properties.

**Figure 4:**
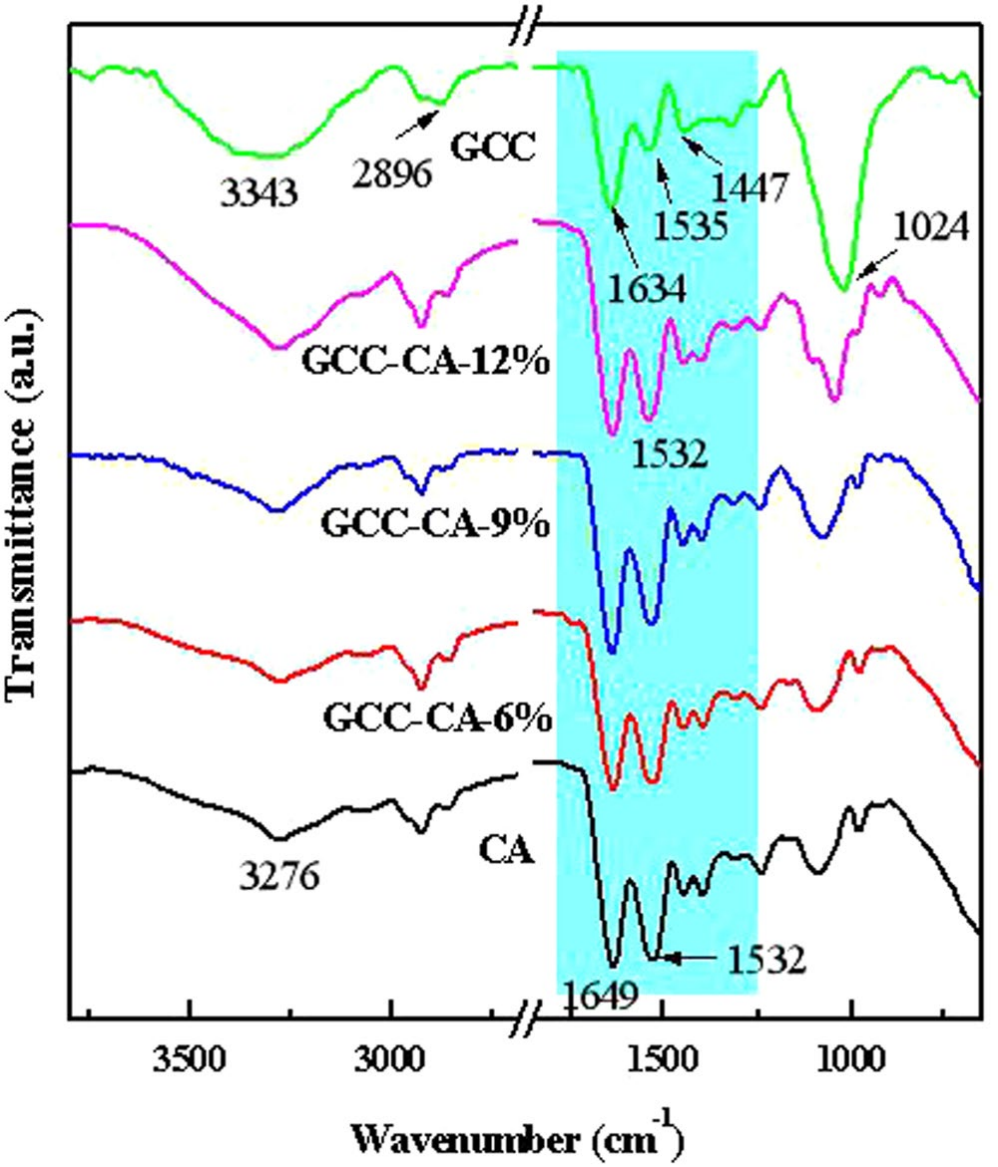
FT-IR spectra of CA-based composite films.

Figure 4 shows the FT-IR spectra of CA-based composite films. For the casein, absorption peaks at 3276, 1649 and 1532 cm^−1^ are attributed to N-H stretching, amide C=O stretching and the bending vibration of N-H (plus C-N stretching)^29^. The characteristic peaks from both casein and GCC are observed in GCC-CA-n, and the O-H stretching vibration bands are around 3343 cm^−1^ for the GCC-CA-6%, GCC-CA-9% and GCC-CA-12%. This broadens and shifts to a lower wavenumber compared with GCC, as a result of new intermolecular interactions formed by the hydrogen bonds between the GCC microgel and CA matrix. In addition, the relative intensity of the vibration bond at 1532 cm^−1^ (-CONH-) in the CA-based films increases and shifts to lower wavenumber, which proved that the intermolecular hydrogen bonds exists between the N-H group from GCC microgel and the -CONH- in the CA matrix^38–40^. The FT-IR spectra of both GCC-CA-6%, GCC-CA-9% and GCC-CA-12% indicated that the composite films consisted of casein and GCC.

**Figure 5:**
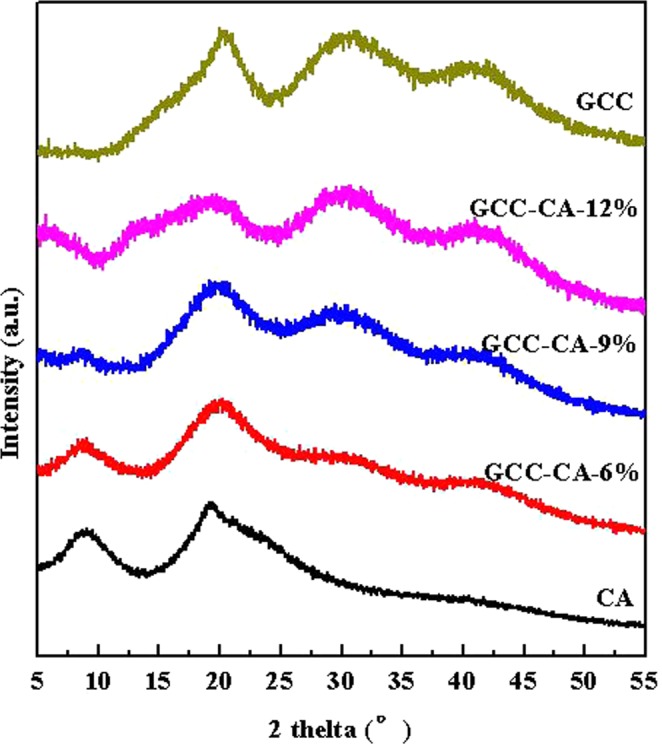
X-ray diffraction patterns of CA, GCC-CA-6%, GCC-CA-9%, GCC-CA-12%, and GCC.

The XRD patterns of CA, GCC and CA-based films are shown in Fig. 5. In the CA diffractogram, there are two clear peaks occurred at 10.8°and 25.6°, showing that casein exhibits some ordered structure. The diffraction angles of the CA-based films are similar to that of the casein film, and in particular, the GCC peaks are not significant in the XRD pattern of the GCC-CA-6%. This can be explained by the low GCC addition in the GCC-CA-n films as well as the crystallinity of the composites is mainly attributable to casein, which denotes that the interaction is physical between casein and GCC microgel. When the GCC content increases GCC peak can be clearly observed, as seen in the diffractograms of CA-based films with 9% and 12% GCC. These more pronounced peaks indicate that the addition of GCC microgel has successfully increased the overall crystallinity of the resulting composite films, implying good compatibility and physical distribution of GCC microgel in the composites^41^.

**Figure 6:**
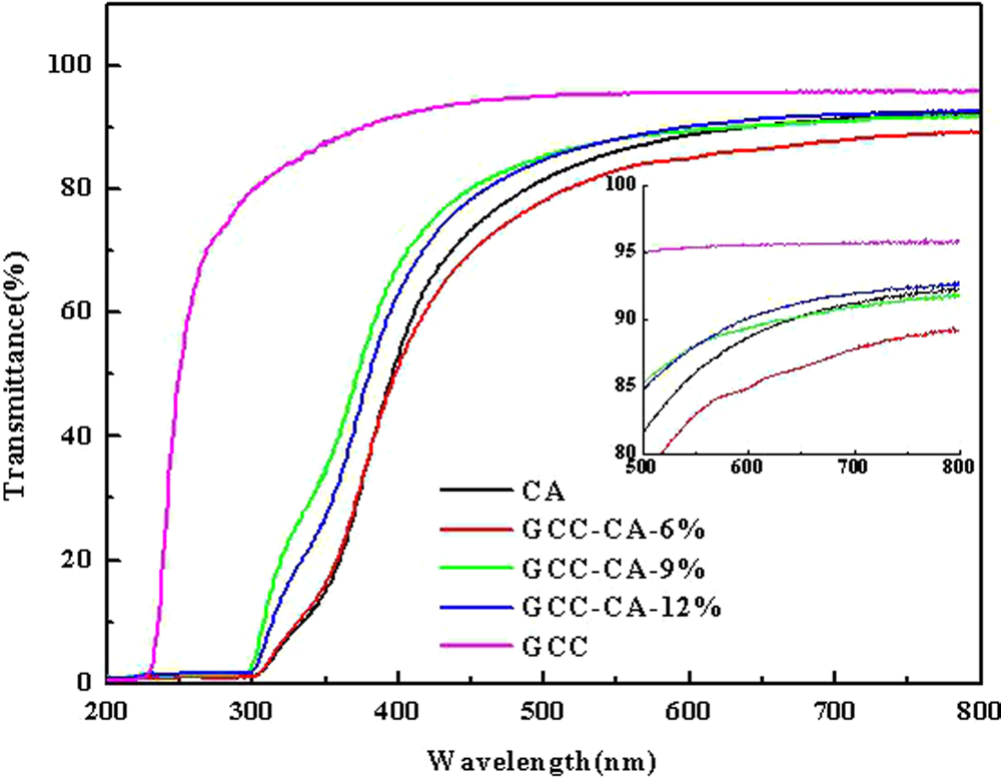
Optical transmittance spectra of CA-based composite films at 200 to 800 nm wavelength.

The light transmittance of the CA, GCC, and CA-based films are shown in Fig. 6. The transmittance (from 500 to 800 nm) for the CA-based films with 9~12% of GCC content are close to each other. The transmittance values for GCC-CA-9% to GCC-CA-12% are higher than 85%, which shows good compatibility between the casein and GCC microgel. The transmittance value of GCC-CA-6% is much lower in the same wavelength range, probably because of the reflection and refraction of part light caused from the relatively rough cross-sections^42^. Interestingly, the transmittance values of the GCC-CA-9% and GCC-CA-12% are about 88% at 550 nm, which was higher than that of casein film, probably due to the strong intermolecular hydrogen bonds between casein and GCC microgel. In addition, for CA-based films, significant UV resistivity occurs in the wavelength range of 200 to 300 nm, indicating that the composite can absorb ultraviolet radiation. It offers potential for the development of UV absorbing materials.

### Thermal stability analysis

**Figure 7:**
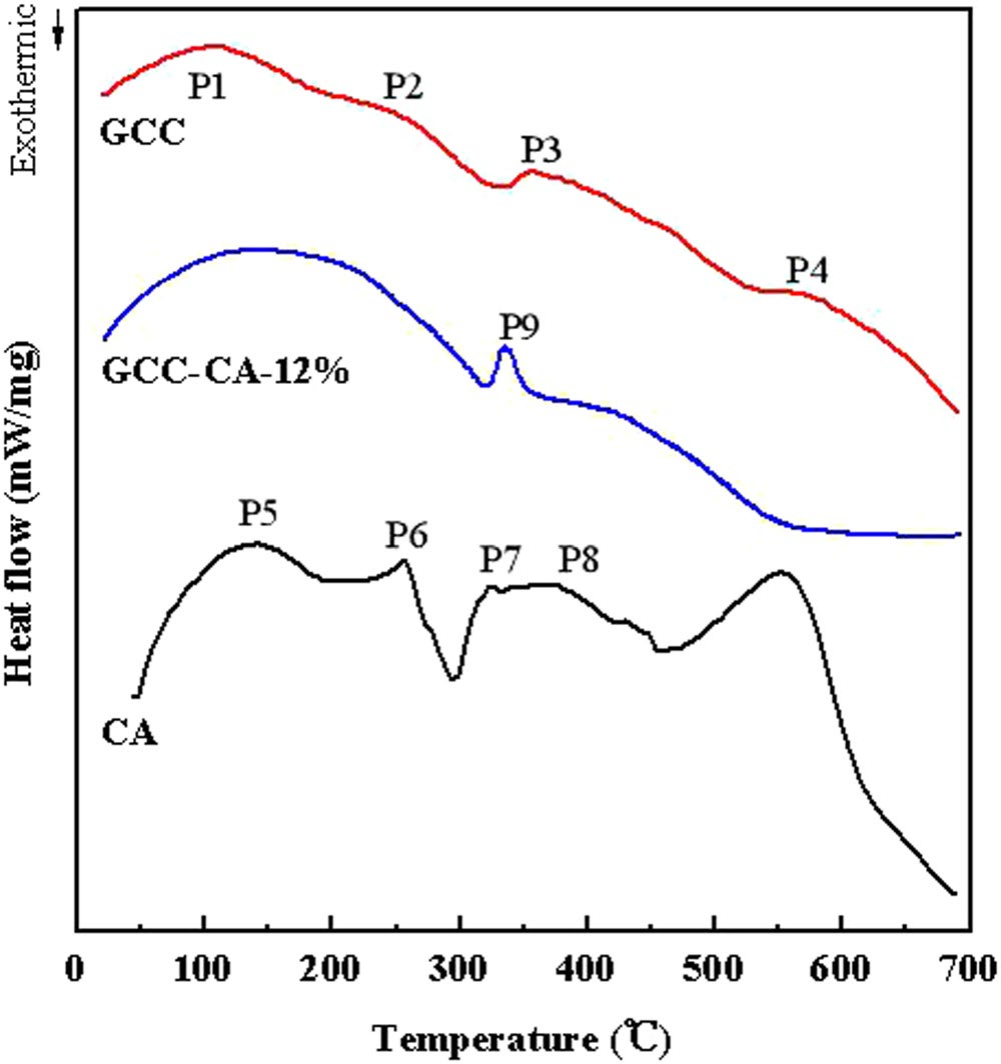
DSC curves of the GCC, CA, and GCC-CA-12% films.

The DSC curves of the CA, GCC, and CA-based films are shown in Fig. 7. The endothermic peaks for P1 (80–130 °C), P2 (260 °C), P3 (355 °C), and P4 (540–566 °C) are the phase transition of the hydrated GCC, cleavage of the GCC crystal, the fracture of hydrogen bonds and van der Waals forces, and charring endothermic in GCC film, respectively. The two endothermic peaks at 117–160 °C (P5) and 255 °C (P6) for casein are attributed to the phase transition of the hydrated casein and cleavage of the casein crystal, respectively. The endothermic peak 7 (322 °C) is associated with the cleavage of hydrogen bonds, van der Waals forces, -s-s bonds, and salt links in casein. Peak 8 (375 °C) is related to the fracture of the C-N, and multiple bonds in the casein, and the peptide bond of the casein had been vaporized in a further thermal decomposition^37^. However, the peak 9 (338 °C) corresponding to the cleavage of the hydrogen bonds for GCC-CA-12% is shifted to higher temperature than that of casein film, indicating the GCC microgel has additional improvement in the thermal stability of casein. This conclusion is different from the results reported by Xie and Kang whose work demonstrated that the addition of microcrystalline cellulose had no obvious influence on the thermal performance of the protein-based films^36,43^. The improvement of thermal stability may be attributed to the strong hydrogen bonds interaction formed between casein and GCC molecular chain due to the abundant polar groups^44^.

### Surface hydrophobicity, moisture absorption and water vapor permeability (WVP) analysis

**Figure 8:**
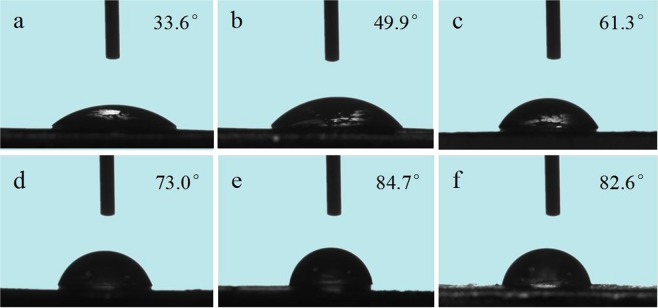
Contact angle images on CA-based film surfaces. (**a**) CA, (**b**) GCC-CA-3%, (**c**) GCC-CA-6%, (**d**) GCC-CA-9%, (**e**) GCC-CA-12%, and (**f**) GCC-CA-15%.

The surface hydrophobicity of CA-based composite films could be determined from the measurement of their water contact angles (WCA). As shown in Fig. 8, casein film presents a low WCA of 33.6°due to its inherent abundant hydrophilic groups. With the addition of GCC, the WCA of the composite films becomes higher. It means that the hydrophobicity of composites is improved. This is due to the structure of CA-based films becomes more homogeneous and compact and a three-dimensional rigid network is formed after incorporating GCC microgel, closing some hydrophilic groups on the CA surface, which prevent water from soaking onto the surface of the films. However, excessive GCC addition (15%) lowered WCA; probably result from the phase separation weakened the interaction between GCC and CA that affect the compatibility.

**Figure 9:**
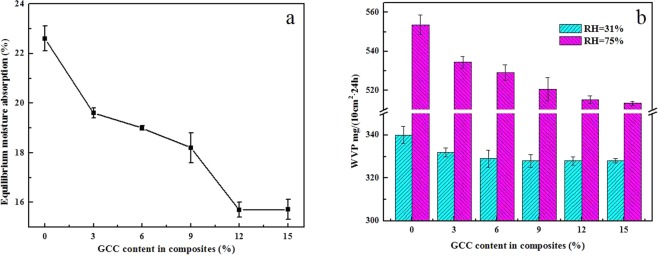
Comparison of equilibrium moisture absorption of the CA-based composite films with different content of GCC (**a**) and the effects of GCC content on the WVP of the CA-based composite films under different relative humidity.

Figure 9a shows the equilibrium moisture absorption of CA and composite films at the relative humidity (RH) of 75%. The CA film has a significantly high moisture absorption amount of 22.6%. In contrast, CA-based composite films demonstrate lower equilibrium moisture absorption. In particular, the equilibrium moisture absorption amount of GCC-CA-12% film is greatly decreased to 15.7%. This can infer that the physical restriction of web-like cellulose in GCC microgel structure towards CA molecule, and the strong hydrogen bonding is formed between GCC and CA matrix, which could result in the low water absorption capability, this is consistent with the result of WCA of CA-based composites. The WVP values of CA, GCC-CA-3%, GCC-CA-6%, GCC-CA-9%, GCC-CA-12%, and GCC-CA-15% are about 553.6, 534.4, 529.2, 520.6, 515.2, 513.4 mg/(10 cm^2^·24 h) at RH of 75%, respectively, which are much higher than those of the 31% RH (Fig. 9b). This suggests that the relative humidity has an important influence on the water vapor permeability. This can be explained by the high humidity causing the dampness pressure difference across the two sides of film is high, water vapor molecules spread quickly on the interior of film, improving the water vapor permeability of film remarkably. However, the WVP of CA-based films is lower than that of pure casein film and decreases with the increasing of GCC microgel content. Xie *et al*.^45^ also reached the same conclusion when studying the WVP of soy protein isolate-based composite films. This is mainly due to the opportunity of adsorption and diffusion for water vapor molecules is restricted by the rigid cellulose and the strong hydrogen bonds interaction between GCC and CA matrix, leading to the lower WVP.

### Mechanical properties

**Figure 10:**
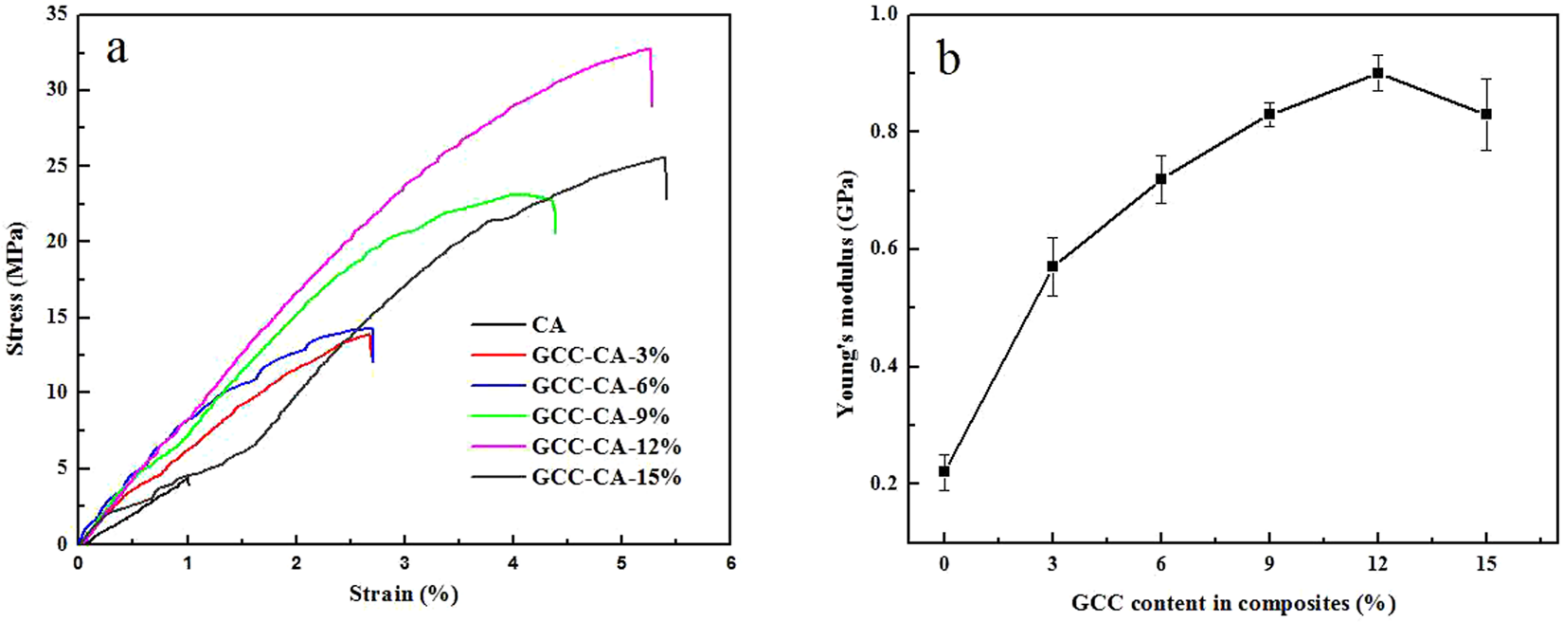
Tensile stress-strain curves of CA, GCC-CA-3%, GCC-CA-6%, GCC-CA-9%, GCC-CA-12%, and GCC-CA-15% films (**a**) and Young’s modulus values of CA-based composite films (**b**).

The stress-strain behaviors of CA-based composite materials are shown in Fig. 10. CA film displays a low tensile strength (σb) of 4.5 MPa and a low breaking elongation (εb) of 1.1%. With the increasing of cellulose microgel solution in the CA matrix, the σb of the CA-based films first increases, and then decreases. The values of σb depict maxima of 32.9 MPa at *W*_GCC_ = 12%, which is about 7 times higher than that of the pure CA (4.5 MPa). Compared with previous report on the nanoparticle enhanced casein-based composite film^46^, the GCC microgel enhanced CA-based film in this work has higher tensile strength. This is due to the relatively strong intermolecular hydrogen bonds in the CA-based composites is formed by the multi-hydroxyl (-OH), amino (-NH_2_) from GCC microgel and the abundant -NH_2_ and carboxyl (-COOH) in CA matrix. The strong interaction gives the CA-based film a higher strength. However, when the GCC microgel content increased is further increased to 15%, the tensile strength is lowered, which could be attributed to the occurrence of severe phase separation when GCC microgel is excessively adds in the CA matrix and the mechanical properties thereof are weakened. These results can also be explained by the cross-sectional morphologies of CA-based composite films. As GCC microgel in the CA matrix increases, the elongation at break of CA-based films increases from 1.1% (CA) to 5.4% (for GCC-CA-15%). The similar results have been reported on cellulose nanocrystal-reinforced soy protein-based films^44^, indicating that the GCC microgel could improve the toughness of CA matrix. The Young’s modulus of the CA-based films significantly increases compared with that of the CA film. This could be ascribed to the enforcing of a rigid cellulose network in GCC microgel. When the GCC addition is further to 15%, the decrease in the Young’s modulus may be due to the phase separation weakens the reinforcement effect. In terms of an overall consideration, the optimal content of GCC microgel in the CA matrix ranges from 10 to 13% and is most suitable for the preparation of CA-based composite films with hydrophobicity, mechanical properties, water vapor permeability and light transmittance.

## Conclusions

In this work, new gelatin-coupled cellulose (GCC) microgel with nano-scale is successfully prepared firstly in NaOH/urea aqueous solution with epichlorohydrin (ECH) as a coupling agent via dialysis and self-dispersion pathway, and then homogenous and compact bio-based CA-based composites are constructed by incorporating GCC microgel in the casein matrix. This work addresses a new way to face such drawbacks with brittleness and sensitive to humidity for casein film. In the composite films, casein and GCC microgel have good compatibility due to the strong hydrogen bonding interactions among the amino groups of the casein, the hydroxyl groups and amino groups of the GCC component. The CA-based composite films have high light transmittance and ultraviolet absorbing ability. Compared with the pure casein, the hygroscopicity of the composite films is significantly reduced and the strength and toughness of the casein matrix are improved. Further, when the GCC content reached 12%, the tensile strength of composite film increases from 4.5 to 32.9 MPa, and the corresponding breaking elongation is improved by 4 times. In addition, incorporating GCC microgel benefits the hydrophobicity and significantly improves the thermal stability of the composite films, as well as exhibits a reduction in water vapor permeability with increase of GCC content at different relative humidity. To this end, using GCC microgel as a reinforcement material to improve the properties of the casein has significant potential for the development of coating, packaging, and bio-film materials.

## Supplementary information

αβΑΒ
