# Supplementary material for: Preparation and characterization of homogeneous and enhanced casein protein-based composite films via incorporating cellulose microgel
Source: Sci Rep. 2019 Feb 4;9:1221. doi: 10.1038/s41598-018-37848-1 (PMC6362078; doi:10.1038/s41598-018-37848-1)
Supplement: Supplementary file 1 — αβΑΒ [file 41598_2018_37848_MOESM1_ESM.pdf]

Supplementary Information

Preparation and characterization of homogeneous and enhanced casein protein-based composite films via incorporating cellulose microgel

Yijun Yao, Hongru Wang, Ruirui Wang & Yong Chai

College of Bioresources Chemical and Materials Engineering, Shaanxi University of Science and Technology, Xi'an 710021, China. Correspondence and requests for materials should be addressed to H.R.W. (email: wanghr@sust.edu.cn).

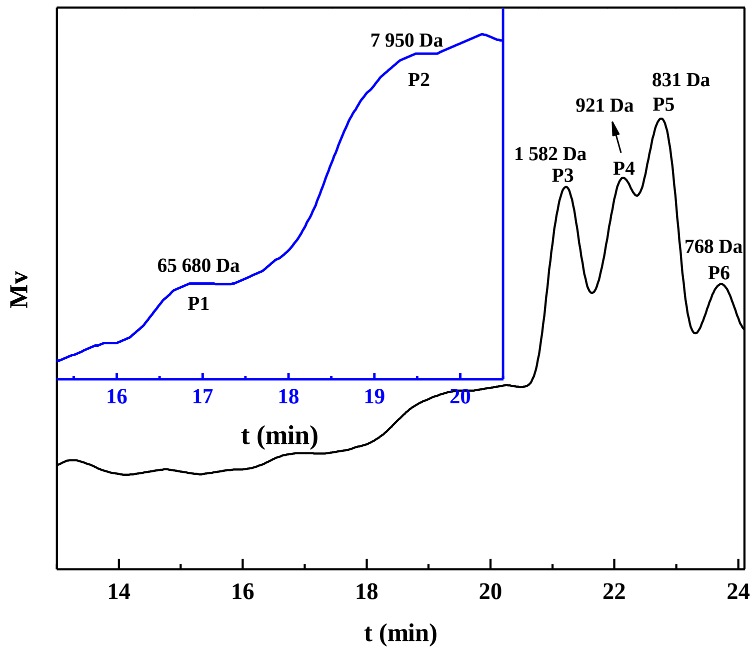

Fig. S1 GPC elution diagrams of native casein.

Table S1

Summary of number- and weight-average molecular weight (Mn and Mw) and polydispersity index (PDI) of casein measured by GPC

Casein	Mn [Da]	Mw [Da]	PDI
Peak 1	64101	65680	1.02
Peak 2	6154	7950	1.29
Peak 3	1558	1582	1.02
Peak 4	877	921	1.05
Peak 5	823	831	1.01
Peak 6	740	768	1.03

Table S2

Summary of number- and weight-average molecular weight (Mn and Mw) and polydispersity index (PDI) of gelatin, Gel-NaOH/urea, C-gel-NaOH/urea, and GCC measured by GPC.

	Mn [kDa]	Mw [kDa]	PDI
Gelatin	0.80~17.74	0.88~63.19	3.561
Gel-NaOH/urea	0.58~1.62	0.60~2.21	1.364
C-gel-NaOH/urea	0.73~3.69	0.78~10.62	2.879
GCC	627.19	636.60	1.015

Note: Gel-NaOH/urea means gelatin treated in NaOH/urea aqueous media at the absence of cellulose and epichlorohydrin; C-gel-NaOH/urea means ECH-coupled gelatin in NaOH/urea aqueous media.

Table S3

The N content (W_N, %), protein content (W_{Pro}, %) and yield (%) of GCC microgel.

	W_{Gel}[a] (%)	W_{Cel}[b] (%)	$n_{ECH}:n_{AGU}$[c]	W_N (%)	W_{Pro} (%)	Yield (%)
Gelatin	100	0	-	14.46	90.38	--
GCC	20	80	2	2.99	18.69	21.3

[a]W_{Gel} = [weight of gelatin/(weight of cellulose + weight of gelatin)] × 100.

[b]W_{Cel} = [weight of cellulose/(weight of cellulose + weight of gelatin)] × 100.

[c]$n_{ECH}:n_{AGU}$ = the molar ratio of epichlorohydrin to anhydroglucose unit of cellulose.

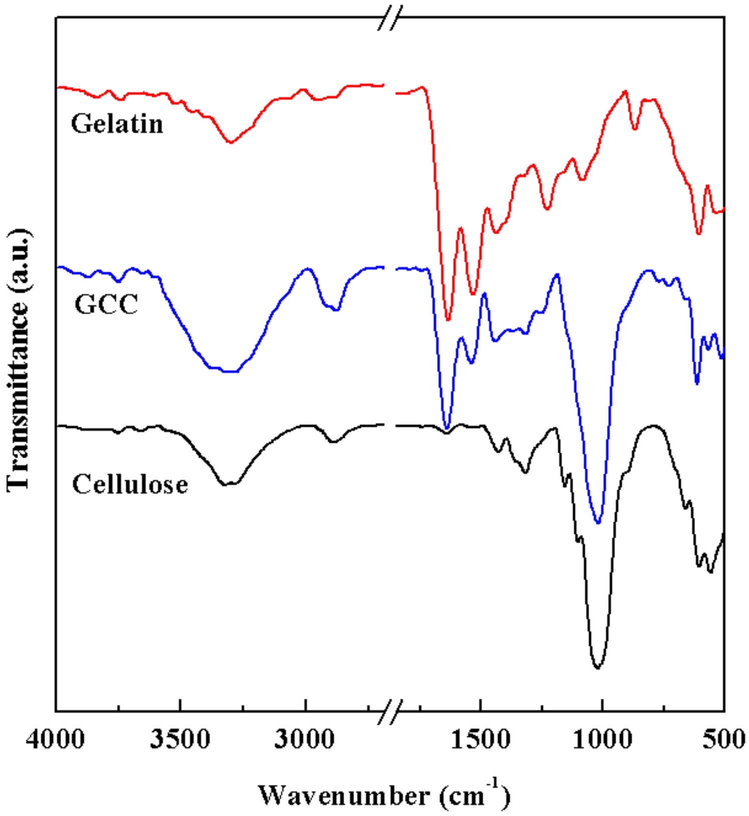

Fig. S2 FTIR of gelatin, cellulose, and GCC microgel.
